# 1,3,5-Tri-*p*-tolyl­pentane-1,5-diol

**DOI:** 10.1107/S160053681400018X

**Published:** 2014-01-11

**Authors:** A. Thiruvalluvar, R. Chithiravel, S. Muthusubramanian, R. J. Butcher

**Affiliations:** a Postgraduate Research Department of Physics, Rajah Serfoji Government College (Autonomous), Thanjavur 613 005, Tamilnadu, India; b Postgraduate Research Department of Chemistry, Rajah Serfoji Government College (Autonomous), Thanjavur 613 005, Tamilnadu, India; c Department of Organic Chemistry, School of Chemistry, Madurai Kamaraj University, Madurai 625 021, Tamilnadu, India; d Department of Chemistry, Howard University, 525 College Street NW, Washington, DC 20059, USA

## Abstract

In the title compound, C_26_H_30_O_2_, the central benzene ring forms dihedral angles of 14.85 (15) and 28.17 (14)° with the terminal benzene rings. The dihedral angle between the terminal benzene rings is 32.14 (13)°. The crystal packing exhibits two strong inter­molecular O—H⋯O hydrogen bonds, forming directed four-membered co-operative rings. A region of disordered electron density, most probably disordered ethyl acetate solvent mol­ecules, occupying voids of *ca* 519 Å^3^ for an electron count of 59, was treated using the SQUEEZE routine in *PLATON* [Spek (2009 ▶). *Acta Cryst.* D**65**, 148–155]. Their formula mass and unit-cell characteristics were not taken into account during refinement. The structure was refined as an inversion twin [absolute structure parameter = −0.3 (4)].

## Related literature   

For the procedure adopted to reduce 1,3,5-tris­(*p*-tol­yl)pentane-1,5-dione, see: Paul *et al.* (2012 ▶). For a less green reported synthesis of the starting diketone, 1,3,5-tris­(*p*-tol­yl)pentane-1,5-dione, see: Yang *et al.* (2005 ▶). For applications of related compounds, see: Sundberg & Faergemann (2008 ▶). For the crystal structures of related compounds, see: Ha & Young (2009 ▶); Barrett *et al.* (2000 ▶). For details of the use of the SQUEEZE and CAVITY routines in PLATON, see: Spek (2009 ▶). For bond-length data, see: Allen *et al.* (1987 ▶).
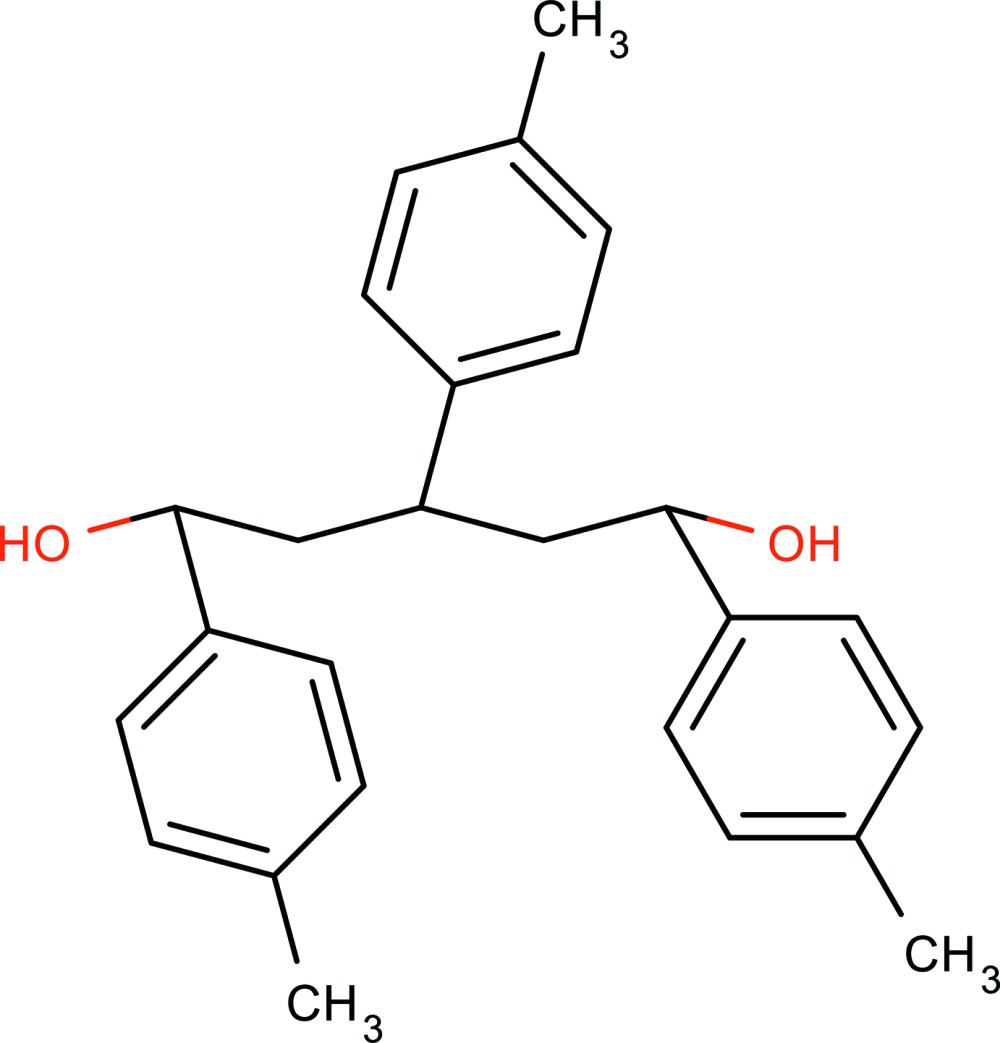


## Experimental   

### 

#### Crystal data  

C_26_H_30_O_2_
*M* *_r_* = 374.50
Trigonal, 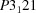

*a* = 14.6205 (5) Å
*c* = 20.2672 (6) Å
*V* = 3751.9 (3) Å^3^
*Z* = 6
Mo *K*α radiation
μ = 0.06 mm^−1^
*T* = 123 K
0.98 × 0.66 × 0.17 mm

#### Data collection  

Agilent Xcalibur Ruby Gemini diffractometer
Absorption correction: analytical [*CrysAlis PRO* (Agilent, 2012 ▶), using a multifaceted crystal model (Clark & Reid, 1995 ▶)] *T*_min_ = 0.957, *T*_max_ = 0.990
35176 measured reflections
7200 independent reflections
5765 reflections with *I* > 2σ(*I*)
*R*_int_ = 0.034

#### Refinement  

*R*[*F* ^2^ > 2σ(*F* ^2^)] = 0.068
*wR*(*F* ^2^) = 0.193
*S* = 1.08
7200 reflections
258 parameters
H-atom parameters constrained
Δρ_max_ = 0.32 e Å^−3^
Δρ_min_ = −0.24 e Å^−3^
Absolute structure: Flack parameter determined using 2073 quotients [(*I* ^+^)−(*I* ^−^)]/[(*I* ^+^)+(*I* ^−^)] (Parsons *et al.*, 2013 ▶)
Absolute structure parameter: −0.3 (4)

### 

Data collection: *CrysAlis PRO* (Agilent, 2012 ▶); cell refinement: *CrysAlis PRO*; data reduction: *CrysAlis PRO*; program(s) used to solve structure: *SIR2011* (Burla *et al.*, 2012 ▶); program(s) used to refine structure: *SHELXL2013* (Sheldrick, 2008 ▶); molecular graphics: *ORTEP-3 for Windows* (Farrugia, 2012 ▶) and *PLATON* (Spek, 2009 ▶); software used to prepare material for publication: *SHELXL2013* and *PLATON*.

## Supplementary Material

Crystal structure: contains datablock(s) global, I. DOI: 10.1107/S160053681400018X/su2686sup1.cif

Structure factors: contains datablock(s) I. DOI: 10.1107/S160053681400018X/su2686Isup2.hkl

Supporting information file. DOI: 10.1107/S160053681400018X/su2686Isup3.cdx

Supporting information file. DOI: 10.1107/S160053681400018X/su2686Isup4.cml

CCDC reference:

Additional supporting information:  crystallographic information; 3D view; checkCIF report

## Figures and Tables

**Table 1:** Hydrogen-bond geometry (Å, °)

*D*—H⋯*A*	*D*—H	H⋯*A*	*D*⋯*A*	*D*—H⋯*A*
O1—H1*A*⋯O5^i^	0.84	1.95	2.786 (3)	174
O5—H5*A*⋯O1^ii^	0.84	1.89	2.716 (3)	170

Symmetry codes: (i) 
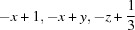
; (ii) 
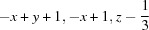
.

**Fig. 1.**
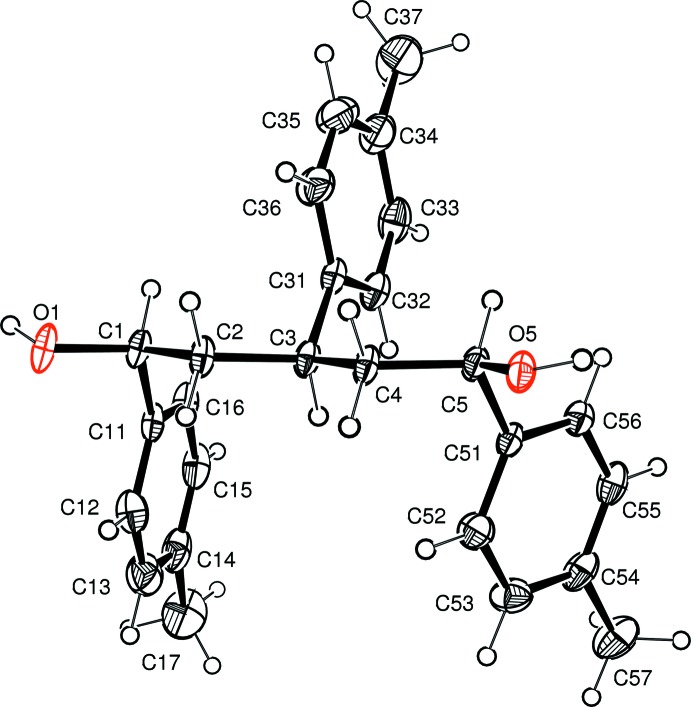
The molecular structure of the title molecule, with atom labelling. Displacement ellipsoids are drawn at the 30% probability level.

**Fig. 2.**
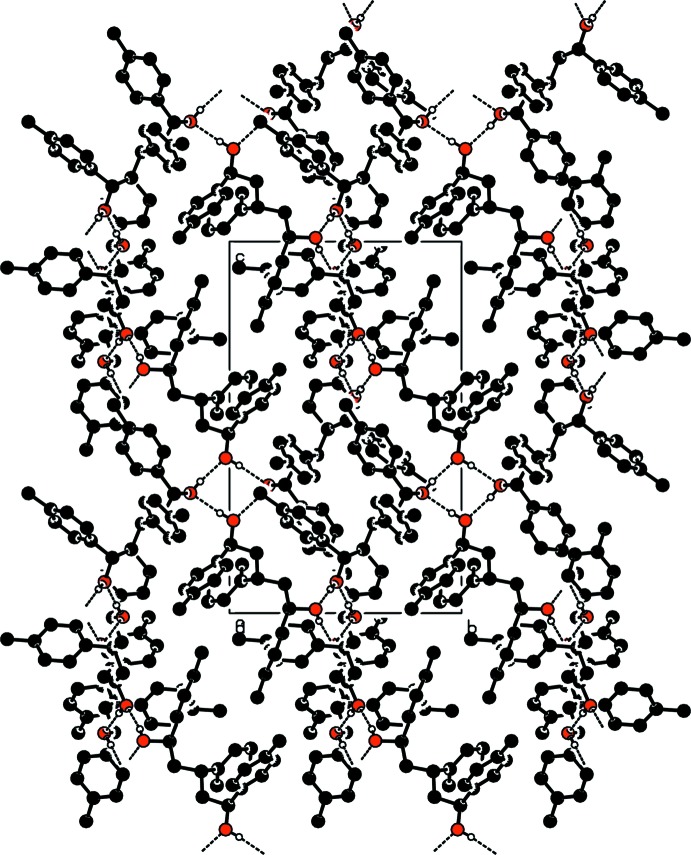
The crystal packing of the title compound, viewed along the a axis. H atoms not involved in hydrogen bonding have been omitted for clarity.

**Fig. 3.**
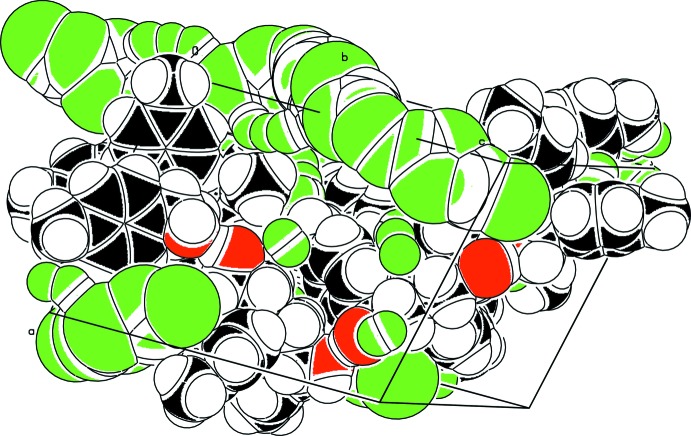
A CavityPlot of the title compound, drawn using the CAVITY routine in PLATON (Spek, 2009), viewed along the b axis. VOIDS in the structure are located and represented by green spheres with radii equal to the contact radius to the nearest van der Waals surface.

## supplementary crystallographic information

### 1. Comment

The synthesis of the title compound has been achieved by the sodium borohydride reduction of the corresponding 1,5-diketone by a method reported recently (Paul *et al.* 2012). The starting diketone, 1,3,5-tris-(*p*-tolyl)pentane-1,5-dione, was prepared by a greener route slightly deviating from the reported one (Yang *et al.* 2005). Though, the separation of the diastereomeric mixture posed problems, it was possible to get one diastereomer in pure form. This acyclic pentane-1,5-diol can be employed for the generation of heterocyclic compounds like tetrahydropyran. Generally, pentane-1,5-diol derivatives are found to be more valuable than several other diols in connection with drug delivery-enhancing potency, pharmaceutical and cosmetic properties, antimicrobial spectrum and toxicity (Sundberg & Faergemann, 2008). The related compounds whose structures have been solved by X-ray diffraction analysis are 2,2,3,3,4,4-hexafluoropentane-1,5-diol (Ha *et al.* 2009) and 3-methylenepentane-1,5-diols (Barrett *et al.* 2000).

In the title molecule, Fig. 1, the pentane-1,5-diol unit (C1—C5/O1/O5) forms a regular zigzag pattern with torsion angles C1—C2—C3—C4 = 178.8 (2)° and C2—C3—C4—C5 = -177.4 (2)°, with the two diol groups pointing in opposite directions. The central benzene ring (C31-C36) forms dihedral angles of 14.85 (15) and 28.17 (14)° with the two terminal benzene rings (C11-C16 and C51-C56, respectively). The dihedral angle between the two terminal benzene rings is 32.14 (13)°. The C—C, C_ar_—C_ar_ and C—O bond lengths are within their normal ranges (Allen *et al.*, 1987).

In the crystal, there are two strong O-H···O hydrogen bonds (Table 1), forming directed 4-membered cooperative O—H···O—H···O—H···O—H rings (Fig. 2). There are large void channels in the crystal structure (Fig. 3) containing residual electron density with high disorder.

### 2. Experimental

To a stirred solution of 1,3,5-tris(*p*-tolyl)pentane-1,5-dione (0.4 g, 1.0 mmol) in methanol, sodium borohydride (0.08 g, 2.2 mmol) was added in portions in ambient conditions. After the completion of the reaction, the mixture was poured onto crushed ice and filtered off. The organic layer was dried over anhydrous sodium sulfate. The diastereomeric mixtures were separated by column chromatography using a mixture of petroleum ether and ethyl acetate (80:20) as eluent. The isolated compound was recrystallized in ethyl acetate to obtain colourless plate-like crystal of the title compound in good yield [0.344 g; 86%].

### 3. Refinement

All H-atoms were positioned geometrically and allowed to ride on their parent atoms: O-H = 0.84 Å, C—H = 0.95, 0.99, 1.00 and 0.98 Å for CH(aromatic), CH_2_, CH and CH_3_ H atoms, respectively, with *U*_iso_(H) = 1.5*U*_eq_(C-methyl and O) and = 1.2*U*_eq_(C) for other H atoms. The disordered solvent molecules occupy ca. 14.3% of the unit-cell volume. This region of disordered electron density, probably disordered ethyl acetate solvent molecules, was treated with the SQUEEZE routine in *PLATON* (Spek, 2009), and the solvent-free model was employed for the final refinement.

### Crystal data

**Table:**

C_26_H_30_O_2_	*D*_x_ = 0.994 Mg m^−^^3^
*M**_r_* = 374.50	Melting point: 373(2) K
Trigonal, *P*3_1_21	Mo *K*α radiation, λ = 0.71073 Å
Hall symbol: P 31 2"	Cell parameters from 10976 reflections
*a* = 14.6205 (5) Å	θ = 3.2–30.9°
*c* = 20.2672 (6) Å	µ = 0.06 mm^−^^1^
*V* = 3751.9 (3) Å^3^	*T* = 123 K
*Z* = 6	Plate, colourless
*F*(000) = 1212	0.98 × 0.66 × 0.17 mm

### Data collection

**Table:**

Agilent Xcalibur Ruby Gemini diffractometer	7200 independent reflections
Radiation source: Enhance (Mo) X-ray Source	5765 reflections with *I* > 2σ(*I*)
Graphite monochromator	*R*_int_ = 0.034
Detector resolution: 10.5081 pixels mm^-1^	θ_max_ = 30.9°, θ_min_ = 3.2°
ω scans	*h* = −20→18
Absorption correction: analytical [*CrysAlis PRO* (Agilent, 2012), using a multifaceted crystal model (Clark & Reid, 1995)]	*k* = −13→19
*T*_min_ = 0.957, *T*_max_ = 0.990	*l* = −28→26
35176 measured reflections	

### Refinement

**Table:**

Refinement on *F*^2^	Secondary atom site location: difference Fourier map
Least-squares matrix: full	Hydrogen site location: inferred from neighbouring sites
*R*[*F*^2^ > 2σ(*F*^2^)] = 0.068	H-atom parameters constrained
*wR*(*F*^2^) = 0.193	*w* = 1/[σ^2^(*F*_o_^2^) + (0.1162*P*)^2^ + 0.2294*P*] where *P* = (*F*_o_^2^ + 2*F*_c_^2^)/3
*S* = 1.08	(Δ/σ)_max_ < 0.001
7200 reflections	Δρ_max_ = 0.32 e Å^−^^3^
258 parameters	Δρ_min_ = −0.24 e Å^−^^3^
0 restraints	Absolute structure: Flack parameter determined using 2073 quotients [(*I*^+^)-(*I*^-^)]/[(*I*^+^)+(*I*^-^)] (Parsons *et al.*, 2013)
Primary atom site location: structure-invariant direct methods	Absolute structure parameter: −0.3 (4)

### Special details

**Table:**

Geometry. Bond distances, angles *etc*. have been calculated using the rounded fractional coordinates. All su's are estimated from the variances of the (full) variance-covariance matrix. The cell e.s.d.'s are taken into account in the estimation of distances, angles and torsion angles

### Fractional atomic coordinates and isotropic or equivalent isotropic displacement parameters (Å^2^)

**Table:**

	*x*	*y*	*z*	*U*_iso_*/*U*_eq_	
O1	0.55511 (15)	0.01657 (13)	0.24921 (8)	0.0403 (5)	
O5	0.53998 (13)	0.37108 (12)	0.01023 (8)	0.0342 (4)	
C1	0.5100 (2)	0.00501 (17)	0.18436 (10)	0.0318 (6)	
C2	0.54021 (19)	0.11650 (16)	0.16162 (10)	0.0317 (6)	
C3	0.49968 (18)	0.12147 (16)	0.09253 (11)	0.0300 (5)	
C4	0.53487 (19)	0.23812 (17)	0.07835 (11)	0.0319 (6)	
C5	0.49703 (18)	0.25811 (17)	0.01230 (10)	0.0302 (5)	
C11	0.5478 (2)	−0.04987 (17)	0.13833 (11)	0.0339 (6)	
C12	0.6544 (2)	−0.0129 (2)	0.12939 (15)	0.0483 (8)	
C13	0.6873 (3)	−0.0652 (3)	0.08777 (17)	0.0600 (10)	
C14	0.6155 (3)	−0.1550 (2)	0.05386 (15)	0.0557 (8)	
C15	0.5101 (3)	−0.1911 (2)	0.06246 (14)	0.0498 (8)	
C16	0.4758 (2)	−0.13936 (19)	0.10424 (12)	0.0404 (7)	
C17	0.6518 (4)	−0.2152 (3)	0.0109 (2)	0.0859 (15)	
C31	0.38070 (19)	0.04976 (17)	0.08632 (11)	0.0322 (6)	
C32	0.3383 (2)	−0.02456 (19)	0.03540 (12)	0.0397 (7)	
C33	0.2302 (3)	−0.0923 (2)	0.03095 (16)	0.0539 (9)	
C34	0.1600 (2)	−0.0889 (3)	0.07530 (16)	0.0571 (9)	
C35	0.2037 (3)	−0.0131 (3)	0.12548 (17)	0.0622 (10)	
C36	0.3115 (2)	0.0543 (2)	0.13062 (14)	0.0481 (8)	
C37	0.0412 (3)	−0.1620 (4)	0.0702 (2)	0.0889 (16)	
C51	0.53006 (17)	0.22032 (16)	−0.04816 (10)	0.0292 (5)	
C52	0.6275 (2)	0.2274 (2)	−0.05428 (13)	0.0433 (7)	
C53	0.6561 (2)	0.1949 (2)	−0.11143 (15)	0.0492 (8)	
C54	0.5862 (3)	0.1539 (2)	−0.16445 (13)	0.0468 (8)	
C55	0.4884 (3)	0.1454 (2)	−0.15807 (12)	0.0451 (8)	
C56	0.46012 (19)	0.17721 (18)	−0.10069 (12)	0.0354 (6)	
C57	0.6178 (3)	0.1197 (3)	−0.22690 (16)	0.0670 (11)	
H1	0.43133	−0.03813	0.18794	0.0382*	
H1A	0.52364	−0.04149	0.26918	0.0605*	
H2A	0.61815	0.16084	0.16195	0.0380*	
H2B	0.51202	0.14717	0.19375	0.0380*	
H3	0.53468	0.09783	0.05974	0.0360*	
H4A	0.50846	0.26475	0.11424	0.0382*	
H4B	0.61299	0.27957	0.07938	0.0382*	
H5	0.41821	0.22284	0.01313	0.0363*	
H5A	0.51238	0.38632	−0.02110	0.0513*	
H12	0.70497	0.04879	0.15198	0.0580*	
H13	0.76073	−0.03920	0.08220	0.0720*	
H15	0.45977	−0.25238	0.03949	0.0598*	
H16	0.40227	−0.16546	0.10955	0.0485*	
H17A	0.59274	−0.26614	−0.01624	0.1289*	
H17B	0.67692	−0.25282	0.03889	0.1289*	
H17C	0.70936	−0.16569	−0.01775	0.1289*	
H32	0.38386	−0.02867	0.00364	0.0476*	
H33	0.20335	−0.14266	−0.00376	0.0647*	
H35	0.15810	−0.00787	0.15675	0.0745*	
H36	0.33833	0.10473	0.16531	0.0577*	
H37A	0.01162	−0.13200	0.03937	0.1333*	
H37B	0.00906	−0.16974	0.11381	0.1333*	
H37C	0.02635	−0.23137	0.05424	0.1333*	
H52	0.67616	0.25504	−0.01868	0.0519*	
H53	0.72368	0.20062	−0.11431	0.0590*	
H55	0.43948	0.11734	−0.19350	0.0542*	
H56	0.39173	0.16933	−0.09732	0.0425*	
H57A	0.69271	0.14053	−0.22448	0.1001*	
H57B	0.60609	0.15363	−0.26511	0.1001*	
H57C	0.57511	0.04281	−0.23148	0.1001*	

### Atomic displacement parameters (Å^2^)

**Table:**

	*U*^11^	*U*^22^	*U*^33^	*U*^12^	*U*^13^	*U*^23^
O1	0.0659 (10)	0.0270 (7)	0.0187 (7)	0.0162 (7)	−0.0097 (7)	0.0014 (6)
O5	0.0541 (8)	0.0381 (7)	0.0228 (7)	0.0324 (5)	0.0016 (6)	0.0036 (6)
C1	0.0456 (11)	0.0261 (9)	0.0183 (9)	0.0138 (8)	−0.0049 (8)	0.0008 (8)
C2	0.0488 (11)	0.0275 (9)	0.0201 (9)	0.0200 (7)	−0.0071 (8)	−0.0015 (8)
C3	0.0446 (10)	0.0301 (9)	0.0172 (9)	0.0201 (7)	−0.0031 (8)	−0.0016 (7)
C4	0.0493 (11)	0.0324 (9)	0.0173 (9)	0.0229 (8)	−0.0018 (8)	0.0010 (8)
C5	0.0408 (10)	0.0349 (9)	0.0200 (9)	0.0227 (7)	0.0022 (8)	0.0040 (8)
C11	0.0524 (12)	0.0316 (9)	0.0208 (10)	0.0233 (8)	−0.0057 (9)	0.0030 (8)
C12	0.0599 (14)	0.0438 (12)	0.0459 (15)	0.0294 (10)	−0.0116 (12)	0.0005 (11)
C13	0.0758 (17)	0.0673 (15)	0.0563 (19)	0.0503 (12)	0.0013 (15)	0.0070 (14)
C14	0.1000 (17)	0.0589 (12)	0.0368 (14)	0.0612 (10)	0.0034 (13)	0.0063 (11)
C15	0.0887 (17)	0.0421 (11)	0.0316 (12)	0.0424 (10)	−0.0113 (12)	−0.0054 (10)
C16	0.0628 (13)	0.0343 (10)	0.0281 (11)	0.0272 (9)	−0.0091 (10)	−0.0010 (9)
C17	0.135 (3)	0.0825 (18)	0.079 (3)	0.0834 (15)	0.012 (2)	−0.0041 (19)
C31	0.0447 (10)	0.0322 (9)	0.0206 (10)	0.0198 (8)	−0.0021 (8)	0.0023 (8)
C32	0.0535 (13)	0.0381 (10)	0.0250 (11)	0.0210 (9)	−0.0023 (10)	−0.0008 (9)
C33	0.0650 (17)	0.0467 (14)	0.0375 (15)	0.0186 (12)	−0.0141 (13)	−0.0031 (11)
C34	0.0486 (15)	0.0633 (18)	0.0431 (15)	0.0157 (13)	−0.0014 (12)	0.0070 (14)
C35	0.0505 (15)	0.081 (2)	0.0465 (17)	0.0265 (14)	0.0079 (13)	0.0019 (16)
C36	0.0528 (14)	0.0574 (14)	0.0322 (13)	0.0262 (11)	−0.0015 (11)	−0.0078 (12)
C37	0.057 (2)	0.095 (3)	0.076 (3)	0.009 (2)	−0.0101 (19)	0.001 (2)
C51	0.0420 (10)	0.0292 (8)	0.0196 (9)	0.0201 (7)	0.0035 (8)	0.0036 (7)
C52	0.0462 (12)	0.0551 (13)	0.0317 (12)	0.0277 (10)	−0.0022 (10)	−0.0068 (11)
C53	0.0489 (12)	0.0564 (14)	0.0455 (15)	0.0287 (10)	0.0068 (11)	−0.0089 (12)
C54	0.0685 (15)	0.0443 (12)	0.0270 (12)	0.0278 (11)	0.0097 (11)	−0.0023 (10)
C55	0.0658 (16)	0.0429 (12)	0.0218 (11)	0.0235 (11)	−0.0053 (11)	−0.0040 (9)
C56	0.0425 (11)	0.0367 (10)	0.0281 (11)	0.0206 (8)	−0.0003 (9)	0.0012 (9)
C57	0.090 (2)	0.0742 (19)	0.0391 (16)	0.0428 (15)	0.0168 (15)	−0.0107 (14)

### Geometric parameters (Å, º)

**Table:**

O1—C1	1.442 (3)	C54—C57	1.515 (5)
O5—C5	1.445 (3)	C55—C56	1.390 (4)
O1—H1A	0.8400	C1—H1	1.0000
O5—H5A	0.8400	C2—H2A	0.9900
C1—C11	1.504 (4)	C2—H2B	0.9900
C1—C2	1.531 (3)	C3—H3	1.0000
C2—C3	1.536 (3)	C4—H4A	0.9900
C3—C4	1.542 (3)	C4—H4B	0.9900
C3—C31	1.522 (4)	C5—H5	1.0000
C4—C5	1.531 (3)	C12—H12	0.9500
C5—C51	1.519 (3)	C13—H13	0.9500
C11—C16	1.386 (3)	C15—H15	0.9500
C11—C12	1.383 (4)	C16—H16	0.9500
C12—C13	1.377 (5)	C17—H17A	0.9800
C13—C14	1.386 (5)	C17—H17B	0.9800
C14—C17	1.511 (6)	C17—H17C	0.9800
C14—C15	1.368 (6)	C32—H32	0.9500
C15—C16	1.386 (5)	C33—H33	0.9500
C31—C32	1.399 (3)	C35—H35	0.9500
C31—C36	1.379 (4)	C36—H36	0.9500
C32—C33	1.386 (5)	C37—H37A	0.9800
C33—C34	1.384 (5)	C37—H37B	0.9800
C34—C35	1.401 (5)	C37—H37C	0.9800
C34—C37	1.521 (6)	C52—H52	0.9500
C35—C36	1.383 (5)	C53—H53	0.9500
C51—C56	1.390 (3)	C55—H55	0.9500
C51—C52	1.381 (4)	C56—H56	0.9500
C52—C53	1.393 (4)	C57—H57A	0.9800
C53—C54	1.395 (4)	C57—H57B	0.9800
C54—C55	1.378 (7)	C57—H57C	0.9800
			
C1—O1—H1A	109.00	C4—C3—H3	108.00
C5—O5—H5A	109.00	C31—C3—H3	108.00
C2—C1—C11	113.19 (19)	C3—C4—H4A	109.00
O1—C1—C2	106.14 (17)	C3—C4—H4B	109.00
O1—C1—C11	111.1 (2)	C5—C4—H4A	109.00
C1—C2—C3	114.33 (17)	C5—C4—H4B	109.00
C2—C3—C31	112.19 (19)	H4A—C4—H4B	108.00
C4—C3—C31	112.4 (2)	O5—C5—H5	109.00
C2—C3—C4	107.39 (17)	C4—C5—H5	109.00
C3—C4—C5	114.92 (18)	C51—C5—H5	109.00
O5—C5—C51	110.92 (17)	C11—C12—H12	120.00
O5—C5—C4	104.57 (17)	C13—C12—H12	120.00
C4—C5—C51	115.1 (2)	C12—C13—H13	119.00
C12—C11—C16	118.7 (3)	C14—C13—H13	119.00
C1—C11—C12	121.0 (2)	C14—C15—H15	120.00
C1—C11—C16	120.3 (3)	C16—C15—H15	120.00
C11—C12—C13	120.1 (3)	C11—C16—H16	120.00
C12—C13—C14	121.4 (4)	C15—C16—H16	120.00
C15—C14—C17	120.3 (3)	C14—C17—H17A	109.00
C13—C14—C15	118.4 (3)	C14—C17—H17B	109.00
C13—C14—C17	121.2 (4)	C14—C17—H17C	109.00
C14—C15—C16	120.9 (3)	H17A—C17—H17B	109.00
C11—C16—C15	120.6 (3)	H17A—C17—H17C	110.00
C3—C31—C36	121.8 (2)	H17B—C17—H17C	109.00
C3—C31—C32	120.4 (2)	C31—C32—H32	120.00
C32—C31—C36	117.8 (3)	C33—C32—H32	120.00
C31—C32—C33	120.5 (3)	C32—C33—H33	119.00
C32—C33—C34	122.2 (3)	C34—C33—H33	119.00
C35—C34—C37	120.9 (3)	C34—C35—H35	119.00
C33—C34—C37	122.5 (3)	C36—C35—H35	119.00
C33—C34—C35	116.5 (3)	C31—C36—H36	119.00
C34—C35—C36	121.7 (4)	C35—C36—H36	119.00
C31—C36—C35	121.3 (3)	C34—C37—H37A	109.00
C52—C51—C56	117.6 (2)	C34—C37—H37B	109.00
C5—C51—C52	123.3 (2)	C34—C37—H37C	110.00
C5—C51—C56	119.2 (2)	H37A—C37—H37B	109.00
C51—C52—C53	121.6 (3)	H37A—C37—H37C	110.00
C52—C53—C54	120.4 (3)	H37B—C37—H37C	109.00
C53—C54—C57	120.4 (4)	C51—C52—H52	119.00
C53—C54—C55	118.1 (3)	C53—C52—H52	119.00
C55—C54—C57	121.5 (3)	C52—C53—H53	120.00
C54—C55—C56	121.1 (3)	C54—C53—H53	120.00
C51—C56—C55	121.2 (3)	C54—C55—H55	119.00
O1—C1—H1	109.00	C56—C55—H55	119.00
C2—C1—H1	109.00	C51—C56—H56	119.00
C11—C1—H1	109.00	C55—C56—H56	119.00
C1—C2—H2A	109.00	C54—C57—H57A	109.00
C1—C2—H2B	109.00	C54—C57—H57B	109.00
C3—C2—H2A	109.00	C54—C57—H57C	109.00
C3—C2—H2B	109.00	H57A—C57—H57B	109.00
H2A—C2—H2B	108.00	H57A—C57—H57C	109.00
C2—C3—H3	108.00	H57B—C57—H57C	109.00
			
O1—C1—C2—C3	179.7 (2)	C12—C13—C14—C15	0.1 (5)
C11—C1—C2—C3	57.6 (3)	C12—C13—C14—C17	−176.9 (3)
O1—C1—C11—C12	−53.8 (3)	C13—C14—C15—C16	−0.2 (5)
O1—C1—C11—C16	125.8 (2)	C17—C14—C15—C16	176.9 (3)
C2—C1—C11—C12	65.4 (3)	C14—C15—C16—C11	−0.1 (4)
C2—C1—C11—C16	−115.0 (3)	C3—C31—C32—C33	177.9 (2)
C1—C2—C3—C4	178.8 (2)	C36—C31—C32—C33	−1.2 (4)
C1—C2—C3—C31	54.8 (3)	C3—C31—C36—C35	−178.3 (3)
C2—C3—C4—C5	−177.4 (2)	C32—C31—C36—C35	0.7 (4)
C31—C3—C4—C5	−53.5 (3)	C31—C32—C33—C34	0.9 (5)
C2—C3—C31—C32	−126.6 (2)	C32—C33—C34—C35	−0.1 (5)
C2—C3—C31—C36	52.4 (3)	C32—C33—C34—C37	179.2 (3)
C4—C3—C31—C32	112.3 (3)	C33—C34—C35—C36	−0.4 (5)
C4—C3—C31—C36	−68.7 (3)	C37—C34—C35—C36	−179.7 (4)
C3—C4—C5—O5	−179.6 (2)	C34—C35—C36—C31	0.1 (5)
C3—C4—C5—C51	−57.6 (3)	C5—C51—C52—C53	−177.9 (2)
O5—C5—C51—C52	80.7 (3)	C56—C51—C52—C53	1.4 (4)
O5—C5—C51—C56	−98.7 (2)	C5—C51—C56—C55	177.4 (2)
C4—C5—C51—C52	−37.8 (3)	C52—C51—C56—C55	−1.9 (3)
C4—C5—C51—C56	142.9 (2)	C51—C52—C53—C54	0.0 (4)
C1—C11—C12—C13	179.0 (3)	C52—C53—C54—C55	−0.9 (4)
C16—C11—C12—C13	−0.6 (4)	C52—C53—C54—C57	179.3 (3)
C1—C11—C16—C15	−179.1 (2)	C53—C54—C55—C56	0.4 (4)
C12—C11—C16—C15	0.5 (4)	C57—C54—C55—C56	−179.8 (3)
C11—C12—C13—C14	0.3 (5)	C54—C55—C56—C51	1.1 (4)

### Hydrogen-bond geometry (Å, º)

**Table:**

*D*—H···*A*	*D*—H	H···*A*	*D*···*A*	*D*—H···*A*
O1—H1*A*···O5^i^	0.84	1.95	2.786 (3)	174
O5—H5*A*···O1^ii^	0.84	1.89	2.716 (3)	170

Symmetry codes: (i) −*x*+1, −*x*+*y*, −*z*+1/3; (ii) −*x*+*y*+1, −*x*+1, *z*−1/3.
